# High Anemia Awareness Does Not Mitigate Insomnia Risk in Ghanaian University Students: A Cross‐Sectional Study

**DOI:** 10.1155/bmri/8968775

**Published:** 2026-10-08

**Authors:** Livingstone Agyapong, Jason Brian Apreku, Isaac Acheampong, Godsway Kpeglo, Obed Nana Kwesi Ansaful, Emmanuella Sagoe, Christiana Efua Dadzie, Akrofi Charlotte Sirbah, Mohammed Awal Yusif, Safianu Apalebilah, Gabriel Owusu, David Mawutor Donkor, Dion Siame, Joseph Boachie

**Affiliations:** ^1^ Department of Medical Laboratory Sciences College of Health and Allied Sciences University of Cape Coast Cape Coast Central Region Ghana ucc.edu.gh; ^2^ Department of Medical Laboratory Science Faculty of Health and Allied Sciences KAAF University Gomoa Fetteh Kakraba Central Region Ghana

**Keywords:** anemia, insomnia, knowledge attitude and practices (KAP), university students

## Abstract

**Background:**

Anemia and insomnia represent significant public health concerns among university students. However, their interrelationship and the potential role of anemia prevention knowledge in mitigating these conditions remain unexplored in Ghanaian tertiary institutions.

**Aim:**

This study determined the prevalence of anemia and insomnia among Ghanaian university students. Likewise, the knowledge, attitudes, and practices regarding anemia prevention and the association between anemia prevention knowledge and insomnia status were explored.

**Methods:**

A cross‐sectional study was conducted among 400 undergraduate students at the University of Cape Coast, Ghana. Hemoglobin levels were measured using a HemoCue analyzer. Structured questionnaires assessed sociodemographic characteristics, anemia knowledge, attitudes, and practices, whereas the Insomnia Severity Index evaluated sleep health. Data were analyzed using chi‐square tests and binary logistic regression.

**Results:**

Anemia prevalence was 34.0%, with females disproportionately affected. Moderate‐to‐severe insomnia affected 13.8% of students. No significant association was found between anemia status and insomnia (*χ* ^2^ = 2.639, *p* = 0.125). Anemia prevention knowledge was generally high (58.8% good knowledge) but showed no protective effect against insomnia (aOR = 1.08, *p* = 0.814). Health‐related faculty students demonstrated superior anemia knowledge across multiple domains compared to nonhealth peers (*p* < 0.001). Age ≥ 25 years independently predicted insomnia (aOR = 4.44, *p* = 0.012). Female sex (aOR = 0.135, *p* < 0.001) and Christian religious affiliation (aOR = 0.298, *p* = 0.018) were significant predictors of anemia.

**Conclusion:**

Anemia and insomnia are prevalent but unrelated conditions among Ghanaian university students. Anemia prevention knowledge alone does not confer protection against insomnia. Targeted interventions should address sex‐specific nutritional vulnerabilities and age‐related sleep disturbances through integrated campus health programs.

## 1. Introduction

Afflicting more than two billion people globally, anemia stands as the most pervasive nutritional deficiency worldwide and a principal contributor to the global burden of disability [1, 2]. The anemia crisis in sub‐Saharan Africa is explicitly illustrated in Ghana. Here, the prevalence rates inflexibly persist at 40.4% among women of reproductive age and 24% among adolescents, signaling critical population‐specific vulnerabilities [3, 4]. Likewise, the anemia burden among Ghanaian university students is profoundly sex‐skewed. Females are reported to exhibit nearly four times the prevalence of their male counterparts (24.1% vs. 6.6%), a situation exacerbated by longitudinal trends showing rates can escalate by over 100% because of academic pressures and inadequate dietary intake [5, 6]. Parallel to the anemia crisis, sleep disorders represent a mounting public health emergency. About 150 million adults are reported to be affected globally, with nearly 16.6% of African and Asian populations reporting severe insomnia symptoms [7]. Within African tertiary institutions, poor sleep quality affects nearly two‐thirds of students (63%), fueled by a triad of chronic illness, academic stress, and presleep electronic device exposure [8].

Despite the concurrent high prevalence of anemia and insomnia in Ghanaian tertiary institutions, no study has examined their interrelationship or explored whether anemia prevention knowledge modulates this association. This is therefore critical oversight given meta‐analytical evidence linking anemia to 30%–40% higher insomnia odds in nonstudent populations [2, 9]. The university environment uniquely amplifies susceptibility to anemia and insomnia through intersecting biological and psychosocial pathways. For instance, menstrual iron losses in females compound the nutritional consequences of poverty‐driven dietary choices, whereas academic stress and lifestyle dysregulation independently undermine sleep architecture [5, 10].

University of Cape Coast′s (UCC) population represents a living cross‐section of Ghana′s ecological and cultural landscape, with students from coastal, forest, and savannah backgrounds. They present with varied dietary habits, traditional food prohibitions, and corresponding anemia susceptibilities which can be examined within a single institutional framework [9]. UCC′s large and diverse undergraduate population (60,406 students) furnishes the statistical power required to detect even modest associations between anemia prevalence, insomnia severity, and prevention knowledge while controlling for multiple confounders [11]. The university′s unique institutional architecture, encompassing both health‐related and nonhealth‐related faculties, enables direct comparison of how formal health curricula shape anemia knowledge and whether such knowledge exerts spillover effects on sleep practices. Complementing this academic diversity, Cape Coast′s urban coastal environment, with its characteristic humidity, overcrowded housing, and nocturnal noise pollution, mirrors conditions across West African coastal cities, enhancing the generalizability of findings to analogous settings.

Likewise, anemia and insomnia are critical health issues; therefore, this study would potentially elevate student health as an urgent policy priority. It may further demonstrate that these interconnected conditions directly jeopardize the cognitive and psychological foundations of academic success in Ghanaian tertiary institutions [8]. If the findings reveal a significant association between anemia and insomnia, it would position anemia as a novel, modifiable risk factor for sleep disturbances, revolutionizing campus health interventions. Conversely, if anemia prevention knowledge proves protective against insomnia independent of anemia status, it would validate health education investments while suggesting psychosomatic pathways linking nutritional literacy to sleep quality. Either outcome would fundamentally reshape how Ghanaian tertiary institutions prioritize and target student health resources.

This study therefore investigated the prevalence of anemia and insomnia among Ghanaian university students, assessed students′ anemia‐related knowledge, attitudes, and preventive practices (KAP), examined relevant sociodemographic differences, and investigated the relationship between anemia status, anemia‐related knowledge, and insomnia status.

## 2. Materials and Methods

### 2.1. Study Design

The study was conducted between July and September 2025 and was carried out among regular undergraduate UCC students. At the time of the study, a well‐structured questionnaire was designed to assess anemia knowledge, misconceptions, and practices. Also, the Insomnia Severity Index (ISI) was used to assess insomnia status. We aimed to determine the prevalence of anemia and insomnia and their role in anemia prevention knowledge.

### 2.2. Study Setting

The study site was UCC, located in Cape Coast, the capital city of the Central Region of Ghana. The university was established in 1962 and has a population of 78,485 from the 2022/2023 academic year statistics, with 60,406 undergraduates. This renowned institution has five colleges. They are the College of Agriculture and Natural Sciences (CANS), College of Education Studies (COES), College of Humanities and Legal Studies (CHLS), and College of Health and Allied Sciences (CoHAS). For our study, we grouped students into health‐related and nonhealth‐related faculties. The health‐related students were from CoHAS.

### 2.3. Study Population and Sampling

In the study, we included regular undergraduate students from either health‐related or nonhealth‐related faculties. Some of the health‐related programs are medical laboratory science, nursing, optometry, biochemistry, nutrition/dietetics, biomedical science, and sport science, whereas mathematics, social science, agricultural science, human resources, accounting, finance, computer science, and chemistry are some of the nonhealth‐related programs. A random sampling technique was employed to select students from various programs at UCC.

### 2.4. Sample Size

Conchran′s formula was used to determine sample size [12]:

n=Z2P1−Pd2.

Where:

• *n* = sample size
• *Z* = *Z* − score corresponding to the desired confidence level (1.96 for 95%)
• *P* = estimated prevalence (set at 0.5 to maximize variability)
• *d* = margin of error (0.05)

Substituting the values into the formula

n=1.962×0.510.5−0.052=384.16.

The minimum required sample size was roughly 384 students.

All 400 students returned and filled all surveys; final sample size was 400.

### 2.5. Inclusion and Exclusion Criteria

#### 2.5.1. Inclusion Criteria

Only consented regular undergraduate students offering either a health‐related or nonhealth‐related study program were recruited for the study.

#### 2.5.2. Exclusion Criteria

Postgraduate, sandwich, and distance education students were not involved in the study.

### 2.6. Data Collection and Laboratory Procedures

#### 2.6.1. Hemoglobin (Hb) Screening

Capillary blood sample was taken by trained and skilled laboratory scientists. The Hb 301 HemoCue analyzer (HemoCue AB, Angelholm, Sweden) was used to test for the Hb. This HemoCue analyzer is a portable and battery‐operated device that has been validated for its accuracy in both clinical and field settings. No fasting was required from the patients before Hb measurement. Anemia classification followed a Hb classification used in a recent study within the same population [6], thresholds were below 11.5 g/dL for females and below 12.5 g/dL for males.

#### 2.6.2. Data Collection Tool

Our study′s structured questionnaire has two tools: the anemia prevention questionnaire and the ISI. (Details of the questionnaire are provided in File S1).

##### 2.6.2.1. Anemia Knowledge Assessment Tool

The anemia prevention knowledge was adopted from the literature and was reconstructed following recommended guidelines [9, 13]. The questionnaire was tailored to suit the university′s local setting. With the help of a team of hematologists, we incorporated questions from local anemia knowledge prevention studies. This section is made up of four: demographic (sex, age, level, faculty, history of knowledge, alcohol use, and multivitamin use), assessment of anemia knowledge (risk factors, causes, and symptoms), misconceptions (cultural and erroneous belief systems), and preventive practices (diet, interventions, and supplements). The questions were multiple‐choice to ensure measurable data. The questionnaire was pretested on 40 participants with a Cronbach′s alpha of 0.7373, showing internal consistency and preliminary validation; no modification was made to the prevalidation.

###### 2.6.2.1.1. Scoring and Classification

The questionnaire domains were scored individually to highlight general KAP, misconceptions, and prevention and management of anemia.

Eight knowledge questions were used to assess the general anemia knowledge. One point was awarded for each correct response and zero points for each wrong response or uncertain answers; the maximum score was 8 points. The mean general knowledge score was 3.84 + 1.93. Those who scored 50% or more of the maximum possible score (≥ 4) were considered to have a good general knowledge, and those who scored 0–3 were considered to have a poor general knowledge. The 50% cutoff was applied in accordance with the approach used in previous literature (Doan et al., 2022; Dorji et al., 2020; J. S. et al., 2021).

The attitudes and practices were evaluated by nine questions. Each item was scored according to the predefined response indicating an appropriate attitude or practice, giving a possible score range of 0–9. The mean score was 5.75 ± 1.60. Participants who scored ≥ 50% of the maximum possible score (≥ 5) were considered to have good attitudes and practices and those who scored 0–4 were considered to have poor attitudes and practices.

Misconceptions regarding anemia were assessed separately using eight questions, with a mean score of 4.81 ± 2.17. The items were scored on the basis of the correctness of the responses; the negatively worded misconception items were reverse‐coded if required to ensure a uniform direction of the scoring. Higher scores reflect more appropriate/correct responses. The possible score range was therefore 0–8, with the 50% set as the cut‐off for classification into the various categories.

Prevention and management of anemia were assessed using 10 questions, with a mean score of 6.25 ± 2.74. One point was awarded for each appropriate/correct answer and zero points for each inappropriate/incorrect answer with a possible range of 0–10 points. Participants with scores of 50% or more of the maximum possible score (categorized as good prevention and management) and those with scores of 0–4 (categorized as poor prevention and management) were identified.

##### 2.6.2.2. ISI Tool

The ISI was embedded within a structured questionnaire that also assessed sociodemographic characteristics, anemia prevention KAP. The ISI was selected based on its established validity and reliability in diverse populations [14]. The original validation study by Bastien et al. [14], demonstrated strong psychometric properties, including high internal consistency (Cronbach^′^s *α* = 0.90–0.91) and good test‐retest reliability. The tool has been widely used in university student populations, supporting its appropriateness for this study context.

Although the study does not report a Cronbach′s alpha for the ISI specifically within this Ghanaian student sample, the tool′s consistent performance across cultural contexts and its standardized administration protocol support the validity of the findings. The reliance on self‐report data represents a methodological consideration, as sleep parameters were not objectively verified through actigraphy or polysomnography; however, the ISI′s focus on perceived sleep quality and daytime impairment aligns with the study′s aim to assess clinically meaningful insomnia symptoms rather than objective sleep architecture.

The ISI is a seven‐item self‐report instrument designed to evaluate the subjective severity of insomnia symptoms. In this study, the tool assessed the following dimensions (Table 1):

**Table 1:** Insomnia severity index (ISI) items and domains assessed.

Item	Domain assessed
1	Severity of difficulty falling asleep
2	Severity of difficulty staying asleep
3	Severity of early morning awakening
4	Satisfaction with current sleep pattern
5	Interference of sleep problems with daytime functioning
6	Noticeability of sleep impairment to others
7	Level of distress caused by sleep difficulties

Each item was rated on a 5‐point Likert scale ranging from 0 to 4, with higher scores indicating greater severity of insomnia symptoms or impairment. The recall period for all items was the preceding 2 weeks, consistent with the standard ISI administration protocol.

Insomnia status was therefore based on participants′ self‐reported symptoms during the preceding 2 weeks and should be interpreted as symptom‐based insomnia severity rather than objectively verified sleep disturbance.

###### 2.6.2.2.1. Scoring and Classification

Total scores were calculated by summing responses across all seven items, yielding a possible range of 0 to 28. Using established cut‐off criteria validated in the original ISI literature, participants were categorized into four severity levels (Table 2):

**Table 2:** Insomnia severity index (ISI) score ranges and severity classifications.

Score range	Classification
0–7	No clinically significant insomnia
8–14	Subthreshold insomnia
15–21	Moderate clinical insomnia
22–28	Severe clinical insomnia

For inferential statistical analyses, participants were dichotomized into two groups:

• No clinically significant insomnia: ISI total score < 15 (combining “no insomnia” and “subthreshold insomnia” categories)
• Insomnia present: ISI total score ≥ 15 (combining “moderate” and “severe” clinical insomnia categories)

This dichotomization strategy was employed to enable binary logistic regression analyses with insomnia status as the dependent variable, facilitating the identification of independent predictors.

### 2.7. Ethical Considerations

Ethical approval was sought from the Institutional Review Board (IRB) of the UCC, Ghana (UCCIRB/CHAS/2025/013). Also, written informed consent was obtained from participants before being recruited into the study. Participants were at liberty to withdraw from the study at any given time.

### 2.8. Statistical Analysis

The study employed a quantitative approach to assess the KAP of participants. A Google form of the questionnaire was used to collect responses. The Hb measurements were entered into the same Microsoft Excel 2016 file where the questionnaire responses were recorded. Cleaning and initial coding were done, and analysis was performed using SPSS version 27. Chi‐square (*χ* ^2^) tests were used to assess the association between anemia knowledge or anemia and insomnia. Binary logistic regression was performed to identify predictors of anemia and insomnia by adjusting for potential confounders like age and sex. The results were tabulated as adjusted odds ratios (aORs) and crude odds ratios (cORs) with 95% confidence intervals (CIs). A *p* − value of 0.05 was used to determine statistical significance. To assess the level of anemia knowledge across the four sections (knowledge, misconceptions, prevention and management, and attitude and practices), they were combined, and each correct response was assigned one point, with total scores ranging from 0 to 35. Participants who scored below the mean of 21 were classified as having poor knowledge, while those who scored 21 or higher were deemed to have good knowledge.

## 3. Results

### 3.1. Sociodemographic Characteristics of Participants

Table 3 presents the distribution of the 400 university students across various sociodemographic and health‐related variables. A majority of the respondents were young, with the largest age group being 18–20 years (39.3%), followed closely by those aged 21–22 years (32.5%). Only 7.8% were aged above 25 years, indicating a youthful student population. Gender distribution was nearly even, with females making up a slight majority (51.2%). Marital status was predominantly single (98.3%), and 94% of respondents identified as Christian. Academically, more than half (55.3%) were in their first year, with lower representations in higher years, particularly fifth‐year students (0.3%). Health‐related programs accounted for 34.5% of the sample, whereas 65.5% were from nonhealth‐related disciplines. In terms of health experiences, 11% reported a history of anemia in the past 3 years, and 7.5% had undergone a blood transfusion. Chronic disease history was rare (3.0%), and a small number (5.8%) admitted to alcohol consumption. Interestingly, a high proportion (64.5%) reported taking iron supplements.

**Table 3:** Sociodemographic characteristics of participants.

Variable	Frequency (%)
Age (years)	
18–20	157 (39.3)
21–22	130 (32.5)
23–25	82 (20.5)
Above 25	31 (7.8)
Gender	
Male	195 (48.8)
Female	205 (51.2)
Marital status	
Single	393 (98.3)
Married	5 (1.3)
Divorced	2 (0.5)
Religion	
Christianity	376 (94.0)
Islam	24 (6.0)
Level of study	
First year	221 (55.3)
Second year	61 (15.3)
Third year	54 (13.6)
Fourth year	62 (15.5)
Fifth year	2 (0.5)
Faculty	
Health related	138 (34.5)
Nonhealth‐related	262 (65.5)
Chronic health status	
Yes	14 (3.5)
No	386 (96.5)
History of anemia in the past 3 years	
Yes	44 (11.0)
No	356 (89.0)
Do you take alcohol?	
Yes	23 (5.8)
No	377 (94.3)
Any history of blood transfusion?	
Yes	30 (7.5)
No	370 (92.5)
Have you ever taken an iron supplement?	
Yes	258 (64.5)
No	142 (35.5)
History of chronic diseases	
Yes	12 (3.0)
No	388 (97.0)

### 3.2. Health Burden of Anemia and Insomnia

Figure 1 shows the prevalence of anemia (Figure 1A) and insomnia among the respondents (Figure 1B). Anemia was identified in 34.0% of students based on Hb levels. Regarding sleep health, an overwhelming proportion (46.3%) reported no clinically significant insomnia symptoms, whereas about 4‐in‐10 (40.0%) experienced subthreshold insomnia. However, moderate clinical insomnia was present in 12.0%, and a small percentage (1.8%) met criteria for severe insomnia. Taken together, 13.8% of participants experienced moderate to severe insomnia symptoms.

**Figure 1:**
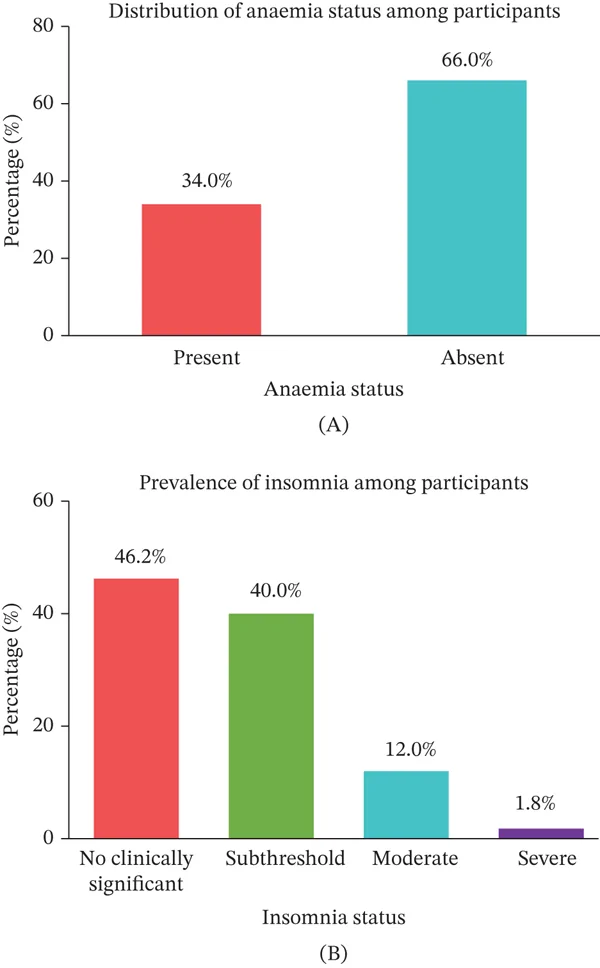
(A) Anemia prevalence based on hemoglobin levels. (B) Distribution of insomnia severity according to Insomnia Severity Index categories.

### 3.3. Knowledge, Misconceptions, Management, and Practices Regarding Anemia by Sex and Faculty

Table 4 presents students′ knowledge, misconceptions, management, and preventive practices regarding anemia by sex and faculty type. Significant differences were observed across multiple variables.

**Table 4:** Association of anemia knowledge, misconceptions, management, and preventive practices with sex and faculty type.

	Total, *n* (%)	Male	Female	*p*	Health related	Nonhealth related	*p*
Knowledge on anemia							
Blood has a reduced ability to carry oxygen when anemia is present				0.131			**< 0.001**
Yes	255 (63.7)	129 (66.2)	126 (61.5)		114 (82.6)	141 (53.8)	
No	34 (8.5)	20 (10.3)	14 (6.8)		7 (5.1)	27 (10.3)	
Not sure	111 (27.8)	46 (23.6)	65 (31.7)		17 (12.3)	94 (35.9)	
Hypotension and loss of appetite are some symptoms of anemia				**0.006**			**< 0.001**
Yes	257 (64.3)	120 (61.5)	137 (66.8)		108 (78.3)	149 (56.9)	
No	35 (8.8)	26 (13.3)	9 (4.4)		6 (4.3)	29 (11.1)	
Not sure	108 (27.0)	49 (25.1)	59 (28.8)		24 (17.4)	84 (32.1)	
Malabsorption and poor diet intake are some causes of anemia				0.063			**< 0.001**
Yes	273 (68.3)	127 (65.1)	146 (71.2)		116 (84.1)	157 (59.9)	
No	27 (6.8)	19 (9.7)	8 (3.9)		6 (4.3)	21 (8.0)	
Not sure	100 (25.0)	49 (25.1)	51 (24.9)		16 (11.6)	84 (32.1)	
Pregnant women are mostly at risk of iron‐deficiency anemia				0.599			**< 0.001**
Yes	209 (52.3)	106 (54.4)	103 (50.2)		94 (68.1)	115 (43.9)	
No	71 (17.8)	35 (17.9)	36 (17.6)		22 (15.9)	49 (18.7)	
Not sure	120 (30.0)	54 (27.7)	66 (32.2)		22 (15.9)	98 (37.4)	
Iron deficiency can cause anemia				0.491			**< 0.001**
Yes	306 (76.5)	146 (74.9)	160 (78.0)		128 (92.8)	178 (67.9)	
No	25 (6.3)	11 (5.6)	14 (6.8)		8 (5.8)	17 (6.5)	
Not sure	69 (17.3)	38 (19.5)	31 (15.1)		2 (1.4)	67 (25.6)	
When untreated anemia could lead to coma				0.528			**< 0.001**
Yes	195 (48.8)	97 (49.7)	98 (47.8)		87 (63.0)	108 (41.2)	
No	30 (7.5)	17 (8.7)	13 (6.3)		10 (7.2)	20 (7.6)	
Not sure	175 (43.8)	81 (41.5)	94 (45.9)		41 (29.7)	134 (51.1)	
Pica occurs as a result of anemia				0.542			**< 0.001**
Yes	101 (25.3)	52 (26.7)	49 (23.9)		50 (36.2)	51 (19.5)	
No	39 (9.8)	16 (8.2)	23 (11.2)		13 (9.4)	26 (9.9)	
Not sure	260 (65.0)	127 (65.1)	133 (64.9)		75 (54.3)	185 (70.6)	
Young children are mostly at risk of iron‐deficiency anemia				**<0.001**			**< 0.001**
Yes	79	38	41		35	44	
No	(19.8)	(19.5)	(20.0)		(25.4)	(16.8)	
Not sure	146 (36.5)	89 (45.6)	57 (27.8)		60 (43.5)	86 (32.8)	
	175 (43.8)	68 (34.9)	107 (52.2)		43 (31.2)	132 (50.4)	
Misconceptions							
Anemia is a spiritual condition				0.205^a^			0.102^a^
Yes	8 (2.0)	7 (3.6)	1 (0.5)		2 (1.4)	6 (2.3)	
No	359 (89.8)	173 (88.7)	186 (90.7)		130 (94.2)	229 (87.4)	
Not sure	33 (8.3)	15 (7.7)	18 (8.8)		6 (4.3)	27 (10.3)	
Anemia is transmissible by body contact				0.553			**< 0.001**
Yes	20 (5.0)	11 (5.6)	9 (4.4)		3 (2.2)	17 (6.5)	
No	326 (81.5)	161 (82.6)	165 (80.5)		131 (94.9)	195 (74.4)	
Not sure	54 (13.5)	23 (11.8)	31 (15.1)		4 (2.9)	50 (19.1)	
Sickle cell anemia affects both males and females equally				0.908			**< 0.001**
Yes	60 (15.0)	30 (15.4)	30 (14.6)		19 (13.8)	41 (15.6)	
No	181 (45.3)	86 (44.1)	95 (46.3)		88 (63.8)	93 (35.5)	
Not sure	159 (39.8)	79 (40.5)	80 (39.0)		31 (22.5)	128 (48.9)	
Women are less prone to iron‐deficiency anemia than men due to menstruation and pregnancy				0.699			**< 0.001**
Yes	36 (9.0)	20 (10.3)	16 (7.8)		11 (8.0)	25 (9.5)	
No	183 (45.8)	87 (44.6)	96 (46.8)		85 (61.6)	98 (37.4)	
Not sure	181 (45.3)	88 (45.1)	93 (45.4)		42 (30.4)	139 (53.1)	
Eating iron‐rich foods daily can help prevent anemia				**0.045**			**< 0.001**
Yes	88 (22.0)	51 (26.2)	37 (18.0)		22 (15.9)	66 (25.2)	
No	175 (43.8)	74 (37.9)	101 (49.3)		84 (60.9)	91 (34.7)	
Not sure	137 (34.3)	70 (35.9)	67 (32.7)		32 (23.2)	105 (40.1)	
Pregnant women may require iron and folic acid supplements even if they eat a balanced diet.				**0.001**			**0.004**
Yes	278 (69.5)	119 (61.0)	159 (77.6)		104 (75.4)	175 (66.4)	
No	50 (12.5)	29 (14.9)	21 (10.2)		21 (15.2)	29 (11.1)	
Not sure	72 (18.0)	47 (24.1)	25 (12.2)		13 (9.4)	59 (22.5)	
Anemia is primarily a disorder of the liver, not the blood				0.629			**< 0.001**
Yes	52 (13.0)	26 (13.3)	26 (12.7)		14 (10.1)	38 (14.5)	
No	235 (58.8)	110 (56.4)	125 (61.0)		107 (77.5)	128 (48.9)	
Not sure	113 (28.2)	59 (30.3)	54 (26.3)		17 (12.3)	96 (36.6)	
Consuming vitamin‐C‐rich foods (like citrus fruits) with meals can help the body absorb iron and prevent anemia				0.711			**< 0.001**
Yes	81 (20.3)	41 (21.0)	40 (19.5)		26 (18.8)	55 (21.0)	
No	187 (46.8)	87 (44.6)	100 (48.8)		86 (62.3)	101 (38.6)	
Not sure	132 (33.0)	67 (34.4)	65 (31.7)		26 (18.8)	106 (40.5)	
Anemia management and prevention							
Blood transfusions can be used to manage severe cases of iron‐deficiency anemia when other treatments are insufficient							**< 0.001**
Yes	185 (46.3)	99 (50.8)	86 (42.0)		91 (65.9)	94 (35.9)	
No	61 (15.3)	25 (12.8)	36 (17.6)		24 (17.4)	37 (14.2)	
Not sure	154 (38.5)	71 (36.4)	83 (40.5)		23 (16.7)	131 (50.0)	
Eating a balanced and iron‐rich diet can prevent anemia				0.090			**< 0.001**
Yes	335 (83.8)	157 (80.5)	178 (86.8)		126 (91.3)	209 (79.8)	
No	16 (4.0)	7 (3.6)	9 (4.4)		7 (5.1)	9 (3.4)	
Not sure	49 (12.3)	31 (15.9)	18 (8.8)		5 (3.6)	44 (16.8)	
Intake of green vegetables, beans, and poultry provides folic acid that can prevent anemia				0.978			**0.020**
Yes	319 (79.8)	155 (79.5)	164 (80.0)		120 (87.0)	199 (76.0)	
No	15 (3.8)	7 (3.6)	8 (3.9)		5 (3.6)	10 (3.8)	
Not sure	66 (16.5)	33 (16.9)	33 (16.1)		13 (9.4)	53 (20.2)	
Treatment of worm infestation can help in anemia management				0.330			**< 0.001**
Yes	189 (47.3)	97 (49.7)	92 (44.9)		79 (57.2)	110 (42.0)	
No	63 (15.8)	33 (16.9)	30 (14.6)		32 (23.2)	31 (11.8)	
Not sure	148 (37.0)	65 (33.3)	83 (40.5)		27 (19.6)	121 (46.2)	
Having regular medical check‐ups helps to monitor and manage anemia				**0.005**			**0.046**
Yes	318 (79.5)	149 (76.4)	169 (82.4)		110 (79.6)	208 (79.4)	
No	40 (10.0)	16 (8.2)	24 (11.7)		19 (13.8)	21 (8.0)	
Not sure	42 (10.5)	30 (15.4)	12 (5.9)		9 (6.5)	33 (12.6)	
Preventing malaria infection can help to prevent anemia				0.907			**< 0.001**
Yes	193 (48.3)	95 (48.7)	98 (47.8)		84 (60.9)	109 (41.6)	
No	90 (22.5)	45 (23.1)	45 (22.0)		29 (21.0)	62 (23.3)	
Not sure	117 (29.3)	55 (28.2)	62 (30.2)		25 (18.1)	92 (35.1)	
Iron and folic acid supplements can help manage anemia in females				**0.045**			**< 0.001**
Yes	319 (79.8)	151 (77.4)	168 (82.0)		125 (90.6)	194 (74.0)	
No	12 (3.0)	3 (1.5)	9 (4.4)		6 (4.3)	6 (2.3)	
Not sure	69 (17.3)	41 (21.0)	28 (13.7)		7 (5.1)	62 (23.7)	
Avoiding the intake of non‐nutritious substances like clay or soil (pica) can help prevent interference with iron absorption and reduce anemia risk				0.062			**< 0.001**
Yes	188 (47.0)	80 (41.0)	108 (52.7)		82 (59.4)	106 (40.5)	
No	102 (25.5)	54 (27.7)	48 (23.4)		38 (27.5)	64 (24.4)	
Not sure	110 (27.5)	61 (31.3)	49 (23.9)		28 (13.0)	92 (35.1)	

*Note:* *p*‐values shown in bold are statistically significant.

^a^Fisher′s exact test.

With respect to sex stratification, females were more likely than males to recognize hypotension and loss of appetite as symptoms of anemia (66.8% vs. 61.5%, *p* = 0.006). Differences also emerged in perceptions of risk groups, where females were more uncertain than males about athletes being at risk (*p* < 0.001). In preventive knowledge, a greater proportion of males believed that eating three times daily could prevent anemia (26.2% vs. 18.0%, *p* = 0.045). On the other hand, females were significantly more aware that pregnant women who eat well still require iron and folic acid supplementation (77.6% vs. 61.0%, *p* = 0.001). In management, more females than males acknowledged regular medical check‐ups (82.4% vs. 76.4%, *p* = 0.005) and the use of iron and folic acid supplements for females (82.0% vs. 77.4%, *p* = 0.045). Attitudinal differences were also seen, with males more likely than females to report preferring a heavy meal once or twice daily because of financial reasons (56.4% vs. 43.9%, *p* = 0.008).

For faculty stratification, disparities were more extensive. Health‐related students demonstrated stronger knowledge: they were more likely to identify anemia as a reduction in blood′s oxygen‐carrying capacity (82.6% vs. 53.8%, *p* < 0.001), and to recognize hypotension and loss of appetite as symptoms (78.3% vs. 56.9%, *p* < 0.001). They also showed better understanding of causes, including malabsorption and poor diet (84.1% vs. 59.9%, *p* < 0.001) and iron deficiency (92.8% vs. 67.9%, *p* < 0.001). More health students identified the aged as a high‐risk group (68.1% vs. 43.9%, *p* < 0.001), and linked anemia with pica (36.2% vs. 19.5%, *p* < 0.001) or the possibility of coma if untreated (63.0% vs. 41.2%, *p* < 0.001). Faculty differences also extended to risk perception, with more health students identifying athletes (25.4% vs. 16.8%, *p* < 0.001). Nonhealth students, however, showed higher levels of misconceptions, being more likely to believe anemia is contagious (6.5% vs. 2.2%, *p* < 0.001), a liver disorder (14.5% vs. 10.1%, *p* < 0.001), or influenced by eating early or thrice daily (*p* < 0.001).

For management and prevention, health students more often cited blood transfusion as a management option (65.9% vs. 35.9%, *p* < 0.001), and recognized preventive strategies such as a balanced iron‐rich diet (91.3% vs. 79.8%, *p* < 0.001), eating vegetables and beans for folate (87.0% vs. 76.0%, *p* = 0.020), treating worm infestation (57.2% vs. 42.0%, *p* < 0.001), preventing malaria (60.9% vs. 41.6%, *p* < 0.001), avoiding clay/pica (59.4% vs. 40.5%, *p* < 0.001), and preventing kidney disorders (55.8% vs. 41.2%, *p* = 0.003). They were also more likely to endorse iron and folic acid supplementation for females (90.6% vs. 74.0%, *p* < 0.001) and avoiding excessive alcohol (73.9% vs. 63.4%, *p* = 0.024). Both groups cited regular check‐ups, but the distribution differed significantly (*p* = 0.046).

Finally, for attitudes and practices, health students were significantly more likely to consult a physician when symptomatic (62.3% vs. 47.3%, *p* = 0.005) and to seek health education (69.6% vs. 43.5%, *p* < 0.001).

### 3.4. Categorization of Anemia Prevention Knowledge and Insomnia Status

Figure 2 summarizes the binary classification of two key variables, insomnia status (Figure 2A) and anemia prevention knowledge (Figure 2B), used in inferential analyses. Based on ISI scores, 86.2% of students were categorized as having no clinically significant insomnia (ISI < 15), whereas 13.8% were classified as experiencing insomnia (ISI ≥ 15), based on established cut‐offs. For knowledge classification, majority (58.8%) of participants had good general knowledge on anemia prevention.

**Figure 2:**
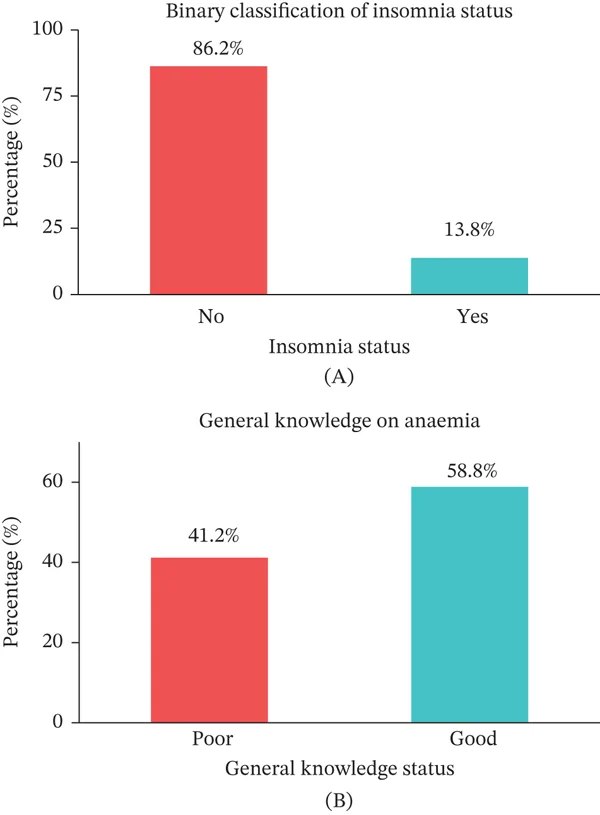
(A) Distribution of insomnia status among university students. (B) Distribution of general knowledge on anemia among university students.

### 3.5. Relationship of Anemia Status, Knowledge as Well as Demographics, With Insomnia Status

Table 5 presents chi‐square test results comparing students with and without insomnia across sociodemographic and health‐related characteristics. There was no significant association between anemia prevalence and insomnia (*χ* ^2^ = 2.639, *p* = 0.125). Anemia prevention knowledge showed no significant relationship with insomnia (*χ* ^2^ = 0.248, *p* = 0.660), suggesting knowledge level alone may not determine sleep health outcomes.

**Table 5:** Association between anemia, sociodemographic characteristics, knowledge, and insomnia status.

Variable	Insomnia status		*x* ^2^	*p*
	No insomnia	Insomniac		
Anemia			2.639	0.125
Present	112 (32.5)	24 (43.6)		
Absent	233 (67.5)	31 (56.4)		
Anemia prevention knowledge				
Poor	144 (41.7)	21 (38.2)	0.248	0.660
Good	201 (58.3)	34 (61.8)		
Age (years)				
18–20	141 (40.9)	16 (29.1)		
21–22	116 (33.6)	14 (25.5)	11.051	0.011^a^
23–25	66 (19.1)	16 (29.1)		
25 or older	22 (6.4)	9 (16.4)		
Gender				
Male	174 (50.4)	21 (38.2)	2.851	0.110
Female	171 (49.6)	34 (61.8)		
Marital status				
Single	340 (98.6)	53 (96.4)		
Married	4 (1.2)	1 (1.8)	2.404	0.362^a^
Divorced	1 (0.3)	1 (1.8)		
Religion				
Christianity	323 (93.6)	53 (96.4)	0.632	0.555^a^
Islam	22 (6.4)	2 (3.6)		
Level of study				
First year	198 (57.4)	23 (41.8)		
Second year	51 (14.8)	10 (18.2)	6.687	0.139
Third year	45 (13.0)	9 (16.4)		
Fourth year	50 (14.5)	12 (21.8)		
Fifth year	1 (0.3)	1 (1.8)		
Faculty of student				
Health related	118 (34.2)	20 (36.4)	0.098	0.762
Non‐health related	227 (65.8)	35 (63.6)		
Chronic health status				
Yes	13 (3.8)	1 (1.8)	0.534	0.703
No	332 (96.2)	54 (98.2)		
History of anemia in past 3 years				
Yes	34 (9.9)	10 (18.2)	3.360	0.100
No	311 (90.1)	45 (81.8)		
Do you take alcohol				
Yes	22 (6.4)	1 (1.8)	1.819	0.227^*α*^
No	323 (93.6)	54 (98.2)		
Any history of blood transfusion				
Yes	27 (7.8)	3 (5.5)	0.358	0.603^a^
No	318 (92.2)	52 (94.5)		
Have you ever taken iron supplement?				
Yes	221 (64.1)	37 (67.3)	0.214	0.654
No	124 (35.9)	18 (32.7)		
Any history of chronic diseases like diabetes?				
Yes	11 (3.2)	1 (1.8)	0.306	0.711^a^
No	334 (96.8)	54 (98.2)		

^a^Fisher′s exact test.

Among the variables examined, age group was significantly associated with insomnia status (*χ* ^2^ = 11.051, *p* = 0.011). Insomnia prevalence increased with age—16.4% of those aged ≥ 25 years were insomniac, compared with 6.4% of those aged 18–20.

Gender differences were not statistically significant (*p* = 0.110), although more females (61.8%) than males (38.2%) experienced insomnia. Similarly, variables such as marital status, religion, year of study, faculty type, and history of anemia, alcohol use, and chronic disease did not show significant differences between insomnia groups (all *p* > 0.05).

Students in nonhealth‐related programs made up the majority of insomniacs (35 of 55, 63.6%), although not statistically significant (*p* = 0.762). Regarding alcohol intake, 54 out of 55 students with insomnia (98.2%) reported not consuming alcohol, with only one student (1.8%) reporting alcohol use—again not statistically significant (*p* = 0.227).

### 3.6. Assessment of the Predictors of Insomnia

Table 6 shows the results of a binary logistic regression assessing associations between anemia prevention knowledge, sociodemographic variables, and insomnia status.

**Table 6:** Predictors of insomnia based on logistic regression analysis.

Variable	No insomnia, *n*(%)	Insomniac, *n*(%)	cOR (95% CI)	*p*	aOR (95% CI)	*p*
General knowledge						
Poor	144 (41.7)	21 (38.2)	1.00			
Good	201 (58.3)	34 (61.8)	1.16 (0.65–2.11)	0.619	1.08 (0.56–2.13)	0.814
Age (years)						
18‐20	141 (40.9)	16 (29.1)				
21–22	116 (33.6)	14 (25.5)	1.06 (0.49–2.27)	0.878	1.00 (0.41–2.36)	> 0.9
23–25	66 (19.1)	16 (29.1)	2.14 (1.00–4.56)	**0.048**	1.92 (0.70–5.24)	0.204
25 or older	22 (6.4)	9 (16.4)	3.61 (1.38–9.07)	**0.007**	4.44 (1.36–14.1)	**0.012**
Gender						
Male	174 (50.4)	21 (38.2)				
Female	171 (49.6)	34 (61.8)	1.65 (0.93–2.99)	0.094	1.70 (0.88–3.38)	0.121
Marital status						
Single	340 (98.6)	53 (96.4)				
Married	4 (1.2)	1 (1.8)	1.60 (0.08–11.1)	0.675	0.43 (0.02–4.22)	0.514
Divorced	1 (0.3)	1 (1.8)	6.42 (0.25–164)	0.191	2.50 (0.08–75.3)	0.555
Religion						
Christianity	323 (93.6)	53 (96.4)				
Islam	22 (6.4)	2 (3.6)	0.55 (0.09–1.96)	0.433	0.54 (0.08–1.99)	0.421
Level of student						
Level 100	198 (57.4)	23 (41.8)				
Level 200	51 (14.8)	10 (18.2)	1.69 (0.73–3.69)	0.202	1.35 (0.52–3.35)	0.518
Level 300	45 (13.0)	9 (16.4)	1.72 (0.71–3.87)	0.203	1.22 (0.43–3.27)	0.694
Level 400	50 (14.5)	12 (21.8)	2.07 (0.94–4.37)	0.063	1.74 (0.62–4.84)	0.291
Level 500	1 (0.3)	1 (1.8)	8.61 (0.33–223)	0.133	5.83 (0.20–169)	0.244
Faculty						
Health related	118 (34.2)	20 (36.4)				
Nonhealth related	227 (65.8)	35 (63.6)	0.91 (0.51–1.67)	0.754	1.31 (0.65–2.73)	0.454
Chronic health status						
Yes	13 (3.8)	1 (1.8)				
No	332 (96.2)	54 (98.2)	2.11 (0.41–38.8)	0.475	2.10 (0.33–41.5)	0.509
History of anemia (past 3 years)						
Yes	34 (9.9)	10 (18.2)				
No	311 (90.1)	45 (81.8)	0.49 (0.23–1.11)	0.071	0.49 (0.21–1.26)	0.122
Take alcohol						
Yes	22 (6.4)	1 (1.8)				
No	323 (93.6)	54 (98.2)	3.68 (0.75–66.5)	0.207	4.65 (0.84–88.6)	0.155
Blood transfusions						
Yes	27 (7.8)	3 (5.5)				
No	318 (92.2)	52 (94.5)	1.47 (0.50–6.32)	0.538	1.75 (0.51–8.34)	0.419
Iron/multivitamin Supplement use						
Yes	221 (64.1)	37 (67.3)				
No	124 (35.9)	18 (32.7)	0.87 (0.46–1.57)	0.644	0.94 (0.46–1.86)	0.858
History of chronic diseases						
Yes	11 (3.2)	1 (1.8)				
No	334 (96.8)	54 (98.2)	1.78 (0.34–32.8)	0.585	1.77 (0.30–34.0)	0.600

*Note:*This table excludes nonsignificant interaction terms. *p*‐values shown in bold are statistically significant.

Abbreviations: aOR, adjusted odds ratio; CI, confidence interval; cOR, crude odds ratio.

Anemia prevention knowledge showed no significant effect on insomnia in either the crude (cOR = 1.16; 95% CI: 0.65–2.11; *p* = 0.619) or adjusted model (aOR = 1.08; 95% CI: 0.56–2.13; *p* = 0.814).

Age was the strongest predictor in both analyses. Students aged 23–25 years had more than twice the crude odds of experiencing insomnia compared with those aged 18–20 (cOR = 2.136; 95% CI: 1.007–4.532; *p* = 0.048), whereas those aged 25 years or older had over three times higher crude odds (cOR = 3.605; 95% CI: 1.419–9.156; *p* = 0.007). After adjusting for other covariates, the effect remained statistically significant for students aged 25 or older, who had 4.5 times the odds of experiencing insomnia compared with the youngest age group (aOR = 4.44; 95% CI: 1.36–14.1; *p* = 0.012).

Gender differences did not approach significance at the crude level (cOR = 1.647; 95% CI: 0.919–2.953; *p* = 0.094) or at the adjusted level (aOR = 1.695; 95% CI: 0.866–3.317; *p* = 0.123). Marital status, religion, level of study, faculty type, anemia history, alcohol intake, blood transfusion, iron supplementation, and chronic disease history showed no significant associations in either the crude or adjusted models (all *p* > 0.05).

Although interaction terms between anemia knowledge and each sociodemographic variable were explored in earlier models, none were statistically significant (*p* > 0.05) and were excluded from this final model for clarity and interpretability.

### 3.7. Binary Logistic Regression Examining the Influence of Sociodemographic and Health‐Related Factors on Anemia Status

Female sex was significantly associated with anemia status. Compared with males, females had significantly lower odds of being nonanemic in both the crude (cOR = 0.151; 95% CI: 0.093–0.245; *p* < 0.001) and adjusted models (aOR = 0.135; 95% CI: 0.078–0.236; *p* < 0.001), corresponding to approximately 7.41 times higher odds of anemia in females than males after adjustment. Age was significantly associated with anemia status in the crude analysis, with students aged 23–25 years (cOR = 1.910; 95% CI: 1.073–3.398; *p* = 0.028) and those above 25 years (cOR = 3.871; 95% CI: 1.413–10.607; *p* = 0.008) having higher odds of being nonanemic than those aged 18–20 years. These associations were not statistically significant after adjustment, with aORs of 1.479 (95% CI: 0.648–3.374; *p* = 0.352) and 2.624 (95% CI: 0.770–8.941; *p* = 0.123), respectively. Religion was also significantly associated with anemia status. Compared with Christian students, Muslim students had lower odds of being nonanemic in both the crude (cOR = 0.411; 95% CI: 0.179–0.945; *p* = 0.036) and adjusted models (aOR = 0.298; 95% CI: 0.110–0.809; *p* = 0.018), corresponding to higher odds of anemia among Muslim students in this sample.

## 4. Discussion

Our study′s population is young, homogeneous Ghanaian students. Out of 400, 71.8% aged 18–22, which shows most students transitioned directly from high school, confirming the overrepresentation of first‐year students [15]. This age range may have an influence on their health because young students are mostly less independent in diet and wellbeing, which possibly can increase the risk of anemia. Females were 205, aligning with female enrollment at most Ghanaian universities and colleges that report up to 64% in the literature [16]. When grouped based on belief system, Christians amounted to 376 (94%), which aligns with the national demography where Christianity dominates in both educational, urban, and rural areas. Single students were 393, which fits the age range and mirrors a survey conducted in Ghana, where over 98% of the population were single [17]. Level 100 students were 221, compared with the other levels. This shows sampling bias and limits students′ exposure to anemia knowledge or practice alongside irregular sleep schedules. Fifth‐year students were the least represented group (0.5%), reflecting the distribution of programs and sampling across the university. Two hundred sixty‐two were nonhealth students; this stands a higher chance of health illiteracy, as these students have low knowledge about diseases such as anemia and insomnia. The use of alcohol and a history of any chronic diseases are health indicators for either anemia or insomnia, but they are very low (5.8% and 3.0%, respectively). The use of alcohol frequency aligns with students at the university attitude toward substance abuse in a local study [18]. Also, most students, thus nearly one‐third of the students, agree to the use of iron supplements, which exceeds the national average of Ghana [19] and may raise the question of whether students have easy access to iron supplements, or whether students are becoming more aware of the importance of supplements, and also whether they are mere multivitamins or iron‐specific, since no measurement was done.

The prevalence of anemia in our study was 34%, which is higher than the Ghanaian national average and is generally similar to a longitudinal study conducted in UCC. This study revealed an increase in anemia from 20% at the start of the academic year to 45.1% by the second semester because of academic stress or poor dietary intake [18]. The 34.0% versus 45.1% could be explained by the timing of data collection; our data were collected before academic stress reached its peak, or a difference in methods, such as using capillary instead of blood samples, which may have caused the prevalence to be up by 10%. Also, another study′s prevalence contradicts our findings; the study recorded 65.5% anemia prevalence [20]. This higher prevalence could attest to the fact that the study was only conducted among women of reproductive age who have female‐specific risk factors and high usage of supplements, and it may be due to the difference in settings of their study, along with the food available. Foods sold on university campuses mostly lack naturally iron‐rich nutrients, such as green leafy vegetables.

Figure 1B shows the distribution of insomnia severity, with 13.8% of students experiencing moderate‐to‐severe insomnia. Similar sleep‐related problems have also been reported among university students in Ghana. Simpong et al. reported sleep difficulties in 48.5% of tertiary students using the Pittsburgh Sleep Quality Index, whereas Dziwornu et al. identified insomnia (23.08%) among the common sleep disorders reported by medical students in Ghana [10, 21]. Insomnia status rates range from 9.4% to 38.2% worldwide among students, with developing countries experiencing higher rates because of financial struggles and poor habitation [22, 23]. Our study′s 13.8% moderate to severe insomnia rate falls within this global range but may also be influenced by certain unmeasured factors, such as noise pollution or financial hardship. The 40.0% subthreshold insomnia rate of our study suggests that a large population is at risk of developing clinical insomnia if not addressed; this necessitates that sleep health programs be run on campuses.

Our findings from Table 4 show females had better anemia knowledge than males, in that females better recognized anemia symptoms such as hypotension and loss of appetite (66.8% vs. 61.5%, *p* = 0.006), which is consistent with a study conducted across sub‐Saharan Africa [16]. Males, on the other hand, had uncertain perceptions of females about sports risk, highlighting the need for health education across genders. Health‐related students displayed higher knowledge across all anaemia domains when grouped by faculty. They also identified key causes, such as iron deficiency (92.8% vs. 67.9%, *p* < 0.001), malabsorption (84.1% vs. 59.9%, *p* < 0.001), older age people being easily at risk (68.1% vs. 43.9%, *p* < 0.001), and athletes (25.4% vs. 16.8%, *p* < 0.001). These findings are consistent with studies conducted in both Malaysia and Turkey, where health students showed greater anemia awareness and preventive practices. [24, 25]. Nonhealth students held more misconceptions, such as anemia being a contagious or transmissible disease (6.5% vs. 2.2%, *p* < 0.001) or anemia being a liver disease (14.5% vs. 10.1%, *p* < 0.001). Health students should better recognize preventive measures, such as the role of iron‐rich diets, deworming, malaria prevention, physician consultation, and alcohol moderation, which confirms the role of education in improving preventive behavior [26].

In Table 5, age was the only variable associated with insomnia (*χ* ^2^ = 11.051, *p* = 0.011), and it is consistent with findings linking older age with prolonged stress and lifestyle changes [27]. Anemia knowledge showed no significant association with insomnia status (*χ* ^2^ = 0.248, *p* = 0.660). The lack of an association between anemia knowledge and insomnia status may stem from how anemia knowledge is measured. The anemia knowledge questionnaire focused on factual recall, such as knowing the causes or symptoms of anemia, rather than a deeper understanding or personal relevance. This approach is likely to fail in capturing how individuals use or relate this knowledge to their own health. As a result, these tools lack the sensitivity to detect indirect effects of anemia knowledge on sleep, such as anxiety, symptom interpretation, or behavior. Consequently, measured anemia knowledge may appear unrelated to insomnia status even when subtle cognitive or behavioral links exist. Also, anemia status and insomnia status were not associated (*χ* ^2^ = 2.639, *p* = 0.125), which challenges the meta‐analysis that found a strong association between the two conditions [14]. This may be due to the younger and healthier nature of the student population, especially the first‐year students, where anemia cases are mostly mild. It may also be psychosocial factors like stress, academic workload, anxiety, depression, and lifestyle behaviors that exert a stronger influence on insomnia than Hb status. Most females recorded insomnia (61.8% vs. 38.2%), reflecting other African studies among students, although sex was not associated with insomnia (*p* = 0.110) [13].

All the other variables, including marital status, religion, year of study, faculty, anemic history, alcohol use, and chronic disease, had no significant associations. The lack of association between other variables and insomnia could be due to homogeneity or limitations in the findings of the chi‐square test to assess complex interactions. Multivariate logistic regression showed that age, specifically older age, was associated with anemia status in the crude analysis, but the associations were not statistically significant after adjustment. Older students (25 years and above) after adjustment had more than four times the odds of insomnia compared with those aged 18–20 years of age (aOR = 4.44, 95% CI: 1.36–14.10, *p* = 0.012). This pattern is consistent with local findings where individuals aged 25 years and above experience greater sleep latency, more disturbances, and worse daytime dysfunction [10]. Older students often contend with a heavier academic load, project demands, financial issues, and uncertainty about life after graduation because of the low employment rate in Ghana; all these can disrupt sleep. Females′ sex showed a higher likelihood of insomnia, although the association lacked statistical significance after adjustment (aOR = 1.70, 95% CI: 0.88–3.38, *p* = 0.121). The direction of effect mirrors established evidence that females experience poorer sleep quality and higher insomnia prevalence in Ghanaian and other African student populations [10]. Anemia prevention knowledge showed no association with insomnia (aOR = 0.1.08, 95% CI:0.56–2.13, *p* = 0.814), suggesting that awareness alone fails to translate into behaviors that protect sleep quality.

Sex and religion were identified as independent predictors of anemia among students from logistic regression in Table 7. Female students had lower odds of being nonanemic than male students (aOR = 0.135, 95% CI: 0.078–0.236, *p* < 0.001), corresponding to approximately 7.41 times higher odds of anemia. This sex difference vividly shows the national survey data from the 2022 Ghana Demographic and Health Survey (GDHS), where there is a high prevalence among women of reproductive age (roughly 40%) as compared with a lower nonanemic proportion in men. It shows the uneven burden on women, likely driven by menstrual iron loss, higher iron needs, and micronutrient gaps [3]. Religion also showed a significant association with anemia status. Students who identified as Muslim had lower chances of being nonanemic as compared with Christian students (aOR = 0.298, 95% Cl. 110–0.809, *p* = 0.018), which suggests a higher anemia burden among Muslims in this sample. This aligns with a study using DHS files, where religion (Muslim) was associated with anemia (aOR = 1.27, 95% Cl 1.11–0.1.46) [28].

**Table 7:** Sociodemographic predictors of anemia based on logistic regression analysis.

Variable	Anemia status		cOR (95% CI)	*p*	aOR (95% CI)	*p*
	Present	Absent				
Age (years)						
18–20 (referent)	67 (49.3)	90 (34.1)				
21–22	41 (30.1)	89 (33.7)	1.616 (0.993–2.629)	0.053	1.243 (0.671–2.307)	0.491
23–25	23 (16.9)	59 (22.3)	1.910 (1.073–3.398)	**0.028**	1.479 (0.648–3.374)	0.352
Above 25	5 (3.7)	26 (9.8)	3.871 (1.413–10.607)	**0.008**	2.624 (0.770–8.941)	0.123
Gender						
Male (referent)	28 (20.6)	167 (63.3)				
Female	108 (79.4)	97 (36.7)	0.151 (0.093–0.245)	**< 0.001**	0.135 (0.078–0.236)	**< 0.001**
Marital status						
Single (referent)	134 (98.5)	259 (98.1)				
Married	1 (0.7)	4 (1.5)	2.069 (0.229–18.700)	0.517	0.957 (0.077–11.825)	0.972
Divorced	1 (0.7)	1 (0.4)	0.517 (0.032–8.337)	0.642	0.255 (0.007–9.534)	0.460
Religion						
Christianity (referent)	123 (90.4)	253 (95.8)				
Islam	13 (9.6)	11 (4.2)	0.411 (0.179–0.945)	**0.036**	0.298 (0.110–0.809)	**0.018**
Level of study						
First year (referent)	83 (61.0)	138 (52.3)				
Second year	21 (15.4)	40 (15.2)	1.146 (0.632–2.075)	0.654	0.912 (0.431–1.930)	0.809
Third year	16 (11.8)	38 (14.4)	1.428 (0.750–2.721)	0.278	1.108 (0.490–2.503)	0.805
Fourth year	16 (11.8)	46 (17.4)	1.729 (0.920–3.249)	0.08	1.215 (0.497–2.968)	0.670
Fifth year	0 (0.0)	2 (0.8)	9.711 (0.011–2.011)	> 0.99	10.321 (0.014–2.819)	>0.99
Faculty of student						
Health‐related (referent)	41 (30.1)	97 (36.7)				
Nonhealth‐related	95 (69.9)	167 (63.3)	0.743 (0.477–1.158)	0.189	0.600 (0.352–1.024)	0.061
Chronic health status						
Yes (referent)	7 (5.1)	7 (2.7)				
No	129 (94.9)	257 (97.3)	1.992 (0.684–5.801)	0.206	4.573 (1.283–16.306)	0.109
History of anemia in the past 3 years						
Yes (referent)	25 (18.4)	19 (7.2)				
No	111 (81.6)	245 (92.8)	2.904 (1.536–5.5493)	**0.001**	2.036 (0.946–4.381)	0.069
Do you take alcohol						
Yes (referent)	4 (2.9)	19 (7.2)				
No	132 (97.1)	245 (92.8)	0.391 (0.130–1.172)	0.094	0.447 (0.133–1.503)	0.193
Any history of blood transfusion						
Yes (referent)	11 (8.1)	19 (7.2)				
No	125 (91.9)	245 (92.8)	1.135 (0.524–2.459)	0.749	1.255 (0.473–3.329)	0.649
Have you ever taken an iron supplement?						
Yes (referent)	98 (72.1)	160 (60.6)				
No	38 (27.9)	104 (39.4)	1.676 (1070–2.625)	**0.024**	0.861 (0.502–1.478)	0.587
Any history of chronic diseases like diabetes?						
Yes (referent)	4 (2.9)	8 (3.0)				
No	132 (97.1)	256 (97.0)	0.970 (0.287–3.280)	0.961	0.991 (0.243–4.037)	> 0.99

*Note:* Outcome coding: 0 = anemia present and 1 = anemia absent. Therefore, ORs > 1 indicate higher odds of being nonanemic, whereas ORs < 1 indicate lower odds of being nonanemic. *p*‐values shown in bold are statistically significant.

## 5. Conclusion

Anemia and insomnia prevalence were significant among university students, but no substantial link was established between these two conditions. Gender and religion were the predictors for anemia, whereas for insomnia, old age was the only predictor. Students at the health‐related faculty excel in anemia knowledge, so female students had better anemia knowledge than male students; these two create a knowledge gap across sex and faculty.

## 6. Limitations

There were several limitations in our study. Our sole reliance on self‐reported data on sensitive issues like the use of alcohol and chronic health status may have led to the fact that they are underreported, and the introduction of recall and social desirability bias. Additionally, insomnia status was assessed using the self‐reported ISI rather than objective measures such as actigraphy or polysomnography. Self‐report may be affected by recall, reporting, and social‐desirability biases and may not fully capture objective sleep characteristics. The study design, thus cross‐sectional design limits causal inference and prevents tracking changes over time. Sampling bias is also present, with an overrepresentation of first‐year students. The absence of a significant association for some variables may reflect sample homogeneity or a lack of an analytic tool to detect a weaker association.

## 7. Recommendations

We recommend that future studies should stratify by faculty and year to examine how their demographics influence anemia or insomnia. This will guide campus interventions like nutritious education and screening. Also, future studies should include biomarkers like ferritin and other validated tools like the Pittsburgh Sleep Quality Index, which would strengthen the analysis of the links between anemia and insomnia. Target interventions like iron supplementation and cognitive‐behavioral therapy education should be instituted in the University Health Service. Low anemia knowledge among males and nonhealth students highlights the need for university‐wide education. Incorporating anemia content into curricula, promoting peer‐led initiatives, and addressing socioeconomic barriers could improve practices and reduce prevalence.

Longitudinal studies are needed to evaluate the sustained impact of such interventions. Future research should also apply multivariate models to assess mediators like stress and diet while developing tailored sleep programs. Since age was the strongest predictor of insomnia, interventions should focus on older students.

## Author Contributions

Livingstone Agyapong: data curation, investigation, methodology, resources, writing—review and editing. Jason Brian Apreku: data curation, investigation, methodology, resources, writing—review and editing. Isaac Acheampong: data curation, investigation, methodology, resources, writing—review and editing. Godsway Kpeglo: data curation, investigation, methodology, resources, writing—review and editing. Obed Nana Kwesi Ansaful: data curation, investigation, methodology, resources, writing—review and editing. Emmanuella Sagoe: data curation, investigation, methodology, resources, writing—review and editing. Christiana Efua Dadzie: data curation, investigation, methodology, resources, writing—review and editing. Akrofi Charlotte Sirbah: formal analysis, investigation, methodology, resources, writing—review and editing. Mohammed Awal Yusif: data curation, investigation, methodology, resources, writing—review and editing. Safianu Apalebilah: data curation, investigation, methodology, writing—review and editing. Gabriel Owusu: data curation, investigation, methodology, writing—review and editing. David Mawutor Donkor: data curation, investigation, methodology, writing—review and editing. Dion Siame: data curation, investigation, methodology, writing—review and editing. Joseph Boachie: conceptualization, project administration, data curation, investigation, methodology, supervision, writing—review and editing. All authors agree to be accountable for the content and conclusions of the article.

## Funding

No funding was received for this manuscript.

## Conflicts of Interest

The authors declare no conflicts of interest.

## Supporting information


**Supporting Information** Additional supporting information can be found online in the Supporting Information section. File S1: This file contains the full study questionnaire, including socio‐demographic information, anemia knowledge and misconception assessments, anemia management and prevention items, attitudes and practices toward anemia, and the Insomnia Severity Index (ISI). It was used to collect data for the current study.

## Data Availability

The data that support the findings of this study are available on request from the corresponding author. The data are not publicly available due to privacy or ethical restrictions.

## References

[1] Gardner W. M. , Razo C. , McHugh T. A. , Hagins H. , Vilchis-Tella V. M. , Hennessy C. , Taylor H. J. , Perumal N. , Fuller K. , Cercy K. M. , Zoeckler L. Z. , Chen C. S. , Lim S. S. , Aali A. , Abate K. H. , Abd-Elsalam S. , Abdurehman A. M. , Abebe G. , Abidi H. , Aboagye R. G. , Abolhassani H. , Aboye G. B. A. , Abtew Y. D. , Accrombessi M. M. K. , Adane D. E. A. , Adane T. D. , Addo I. Y. , Adesina M. A. , Adeyinka D. A. , Adnani Q. E. S. , Afzal M. S. , Afzal S. , Agustina R. , Ahinkorah B. O. , Ahmad A. , Ahmad S. , Ahmadi S. , Ahmed A. , Ahmed Rashid T. , Aiman W. , Ajami M. , Akbarialiabad H. , Alahdab F. , al-Aly Z. , Alam N. , Alemayehu A. , Alhassan R. K. , Ali M. A. , Almustanyir S. , al-Raddadi R. M. , al-Rifai R. H. , Altirkawi K. A. , Alvand S. , Alvis-Guzman N. , Amer Y. S. A. D. , Ameyaw E. K. , Amu H. , Anagaw T. F. , Ancuceanu R. , Anoushirvani A. A. , Antwi M. H. , Anvari D. , Arabloo J. , Aravkin A. Y. , Ariffin H. , Aripov T. , Arja A. , Arndt M. B. , Arulappan J. , Aruleba R. T. , Ashraf T. , Asresie M. B. , Athari S. S. , Atlaw D. , Aujayeb A. , Awoke A. A. , Awoke M. A. , Azadnajafabad S. , Azangou-Khyavy M. , Badawi A. , Badiye A. D. , Baghcheghi N. , Bagheri N. , Bagherieh S. , Baig A. A. , Banach M. , Banik P. C. , Bantie A. T. , Barr R. D. , Barrow A. , Bashiri A. , Basu S. , Batiha A. M. M. , Begum T. , Belete M. A. , Belo L. , Bensenor I. M. , Berhie A. Y. , Bhagavathula A. S. , Bhardwaj N. , Bhardwaj P. , Bhat A. N. , Bhutta Z. A. , Bikbov B. , Billah S. M. , Birara S. , Bishai J. D. , Bitaraf S. , Boloor A. , Botelho J. S. B. , Burkart K. , Calina D. , Cembranel F. , Chakraborty P. A. , Chanie G. S. , Chattu V. K. , Chien J. H. , Chukwu I. S. , Chung E. , Criqui M. H. , Cruz-Martins N. , Dadras O. , Dagnew G. W. , Dai X. , Danawi H. A. , Dandona L. , Dandona R. , Darwesh A. M. , das J. K. , das S. , de la Cruz-Góngora V. , Demisse F. W. , Demissie S. , Demsie D. G. , Desai H. D. , Desalegn M. D. , Dessalegn F. N. , Dessie G. , Dharmaratne S. D. , Dhimal M. , Dhingra S. , Diaz D. , Didehdar M. , Dirac M. A. , Diress M. , Doaei S. , Dodangeh M. , Doku P. N. , Dongarwar D. , Dora B. T. , Dsouza H. L. , Edinur H. A. , Ekholuenetale M. , Elagali A. E. M. , Elbahnasawy M. A. , Elbarazi I. , ElGohary G. M. T. , Elhadi M. , el-Huneidi W. , Elmonem M. A. , Enyew D. B. , Eshetu H. B. , Ewald S. B. , Ezzeddini R. , Fagbamigbe A. F. , Fasanmi A. O. , Fatehizadeh A. , Fekadu G. , Feyisa B. R. , Fischer F. , Fitzgerald R. , Foroutan M. , Fowobaje K. R. , Gadanya M. A. , Gaidhane A. M. , Gaihre S. , Gaipov A. , Galali Y. , Galehdar N. , Garg P. , Garg T. , Gebremariam Y. H. , Gebremedhin K. B. , Gebremichael B. , Gela Y. Y. , Gerema U. , Getacher L. , Ghaffari K. , Ghafourifard M. , Ghamari S. H. , Ghasemi Nour M. , Ghashghaee A. , Gholamalizadeh M. , Ghozy S. , Gizaw A. T. T. , Glasbey J. C. , Golechha M. , Goleij P. , Golitaleb M. , Goulart A. C. , Goyomsa G. G. , Guadie H. A. , Gubari M. I. M. , Gudisa Z. , Gunawardane D. A. , Gupta R. , Gupta R. D. , Gupta S. , Gupta V. K. , Guta A. , Habibzadeh P. , Hamidi S. , Handal A. J. , Hanif A. , Hannan M. A. , Harapan H. , Harorani M. , Hasaballah A. I. , Hasan M. M. , Hasani H. , Hassankhani H. , Hassen M. B. , Hay S. I. , Hayat K. , Heidari G. , Hess S. Y. , Heyi D. Z. , Hezam K. , Hiraike Y. , Holla R. , Hossain S. J. , Hosseini K. , Hosseini M. S. , Hosseinzadeh M. , Hostiuc M. , Hostiuc S. , Huang J. , Hussain S. , Hussien F. M. , Ibitoye S. E. , Ilesanmi O. S. , Ilic I. M. , Immurana M. , Inbaraj L. R. , Islam S. M. S. , Ismail N. E. , Jamshidi E. , Janodia M. D. , Jayarajah U. , Jayaram S. , Jebai R. , Jemal B. , Jeyakumar A. , Jha R. P. , Jonas J. B. , Joseph N. , Jozwiak J. J. , Kabir A. , Kalankesh L. R. , Kalhor R. , Kamal V. K. , Kandel H. , Kanko T. K. , Karaye I. M. , Kashoo F. Z. , Katoto P. D. M. C. , Kauppila J. H. , Kaur H. , Kayode G. A. , Kebede A. D. , Keshri V. R. , Keykhaei M. , Khader Y. S. , Khajuria H. , Khalid N. , Khammarnia M. , Khan I. A. , Khan M. A. B. , Khatab K. , Khazaei Z. , Khubchandani J. , Kim Y. J. , Kimokoti R. W. , Kisa S. , Kompani F. , Kosen S. , Koulmane Laxminarayana S. L. , Krishan K. , Kuate Defo B. , Kuddus M. , Kumar G. A. , Kumar N. , Kumar N. , Kurmi O. P. , Kuti O. , Lal D. K. , Landires I. , Larsson A. O. , Lassi Z. S. , Latief K. , Laxmaiah A. , Ledda C. , Lee S. W. , Legesse S. M. , Liu X. , Lorenzovici L. , Machado V. S. M. , Mahajan P. B. , Mahjoub S. , Mahmoodpoor A. , Mahmoudi E. , Malakan Rad E. , Mallhi T. H. , Malta D. C. , Masoudi S. , Masoumi S. Z. , Medina J. R. C. , Mejia-Rodriguez F. , Mendes J. J. M. , Mendoza W. , Mendoza-Cano O. , Mentis A. F. A. , Meresa H. A. , Mestrovic T. , Miazgowski T. , Mirghafourvand M. , and Mirica A. , Prevalence, Years Lived With Disability, and Trends in Anaemia Burden by Severity and Cause, 1990–2021: Findings From the Global Burden of Disease Study 2021, Lancet Haematology. (2023) 10, no. 9, e713–e734, 10.1016/S2352-3026(23)00160-6, 37536353. PMC10465717 37536353

[2] Neumann S. N. , Li J.-J. , Yuan X.-D. , Chen S.-H. , Ma C.-R. , Murray-Kolb L. E. , Shen Y. , Wu S. L. , and Gao X. , Anemia and Insomnia: A Cross-Sectional Study and Meta-Analysis, Chinese Medical Journal. (2021) 134, no. 6, 675–681, 10.1097/CM9.0000000000001306, 33725707. PMC7989983 33725707

[3] Agulu G. G. , Ahissou N. C. A. , Kamiya Y. , Baiden F. , and Matsui M. , Anaemia Prevalence and Risk Factors Among Nonpregnant and Pregnant Women of Reproductive Age in Ghana: An Analysis of the Ghana Demographic and Health Survey Data, Tropical Medicine and Health. (2025) 53, no. 1, 10.1186/s41182-025-00792-8, 40867006. PMC12382169 40867006

[4] Gosdin L. , Tripp K. , Mahama A. B. , Quarshie K. , Amoaful E. F. , Selenje L. , Sharma D. , Jefferds M. E. , Sharma A. J. , Whitehead R. D. , Suchdev P. S. , Ramakrishnan U. , Martorell R. , and Addo O. Y. , Predictors of Anaemia Among Adolescent Schoolchildren of Ghana, Journal of Nutritional Science. (2020) 9, 10.1017/jns.2020.35, 32983426. PMC7503182 32983426

[5] Amoah S. , Yartey A. N. , Fosu Adjei P. , Owusu-Akyaw M. , Boachie J. , Simpong D. L. , and Adu P. , HBV Infection Is an Intermediate-Risk Disease, Whereas Anaemia Is a Mild-to-Moderate Public Health Problem in Young Ghanaian Adults: A Four-Year Retrospective Analysis of Students′ Medical Records, BioMed Research International. (2023) 2023, no. 1, 9318984, 10.1155/2023/9318984, 37475793. PMC10356218 37475793

[6] Amoaning R. E. , Amoako E. S. , Kyiire G. A. , Owusu D. D. , Bruce H. , Simpong D. L. , and Adu P. , Anaemia Prevalence More Than Doubles in an Academic Year in a Cohort of Tertiary Students: A Repeated-Measure Study in Cape Coast, Ghana, Advances in Hematology. (2022) 2022, no. 1, 4005208, 10.1155/2022/4005208. PMC8800615 35103063

[7] Stranges S. , Tigbe W. , Gómez-Olivé F. X. , Thorogood M. , and Kandala N. B. , Sleep Problems: An Emerging Global Epidemic? Findings From the INDEPTH WHO-SAGE Study Among More Than 40,000 Older Adults From 8 Countries Across Africa and Asia, Sleep. (2012) 35, no. 8, 1173–1181, 10.5665/sleep.2012, 22851813. PMC3397790 22851813

[8] Nakie G. , Takelle G. M. , Rtbey G. , Andualem F. , Tinsae T. , Kassa M. A. , Tadesse G. , Fentahun S. , Wassie Y. A. , Segon T. , Kibralew G. , and Melkam M. , Sleep Quality and Associated Factors Among University Students in Africa: A Systematic Review and Meta-Analysis Study, Frontiers in Psychiatry. (2024) 15, 1370757, 10.3389/fpsyt.2024.1370757, 38559402. PMC10979362 38559402

[9] Awuah R. B. , Colecraft E. K. , Wilson M. L. , Adjorlolo L. K. , Lambrecht N. J. , Nyantakyi-Frimpong H. , and Jones A. D. , Perceptions and Beliefs About Anaemia: A Qualitative Study in Three Agroecological Regions of Ghana, Maternal & Child Nutrition. (2021) 17, no. 4, e13181, 10.1111/mcn.13181, 33780144. PMC8476426 33780144

[10] Simpong D. L. , Bockarie A. , Kumah A. B. , Atta-Owusu A. B. , Eshun M. , Frimpong B. A. , Bachella B. , and Osei G. N. , Poor Sleep Quality Remains a Major Challenge Among Tertiary Education Students in Ghana: A Cross-Sectional Study in a Ghanaian University, IBRO Neuroscience Reports. (2025) 18, 130–134, 10.1016/j.ibneur.2024.12.014, 39877590. PMC11773084 39877590

[11] Bashir S. , Ansari A. H. , and Sultana A. , Knowledge, Attitude, and Practice on Antenatal Care Among Pregnant Women and Its Association With Sociodemographic Factors: A Hospital-Based Study, Journal of Patient Experience. (2023) 10, 23743735231183578, 10.1177/23743735231183578, 37377764. PMC10291858 37377764

[12] Chaokromthong K. and Sintao N. , Sample Size Estimation Using Yamane and Cochran and Krejcie and Morgan and Green Formulas and Cohen Statistical Power Analysis by G∗ Power and Comparisions, Apheit International Journal of Interdisciplinary Social Sciences and Technology. (2021) 10, no. 2, 76–86.

[13] Marías Y. and Glasauer P. , Guidelines for Assessing Nutrition-Related Knowledge, Attitudes and Practices, 2014, Food and Agriculture Organization of the United Nations.

[14] Bastien C. H. , Vallières A. , and Morin C. M. , Validation of the Insomnia Severity Index as an Outcome Measure for Insomnia Research, Sleep Medicine. (2001) 2, no. 4, 297–307, 10.1016/S1389-9457(00)00065-4, 11438246. 11438246

[15] Attuquayefio S. and Addo H. , Gender and age Comparison of Information Communication and Technology Usage Among Ghanaian Higher Education Students, American Journal of Information Systems. (2016) 4, no. 1, 1–6.

[16] Shafina A. , Exploring the Gender Differences in Selection of Subjects at Higher Education Levels, International Education Journal: Comparative Perspectives. (2020) 19, no. 2, 87–105.

[17] Baataar C. and Amadu M. , Attitude Towards Marriage and Family Formation Among Ghanaian Tertiary Students: A Study of University for Development Studies, Journal of Asian Development Studies. (2014) 3, no. 2, 122–134.

[18] Boakye H. , Atabila A. , Hinneh T. , Ackah M. , Ojo-Benys F. , and Bello A. I. , The Prevalence and Determinants of Non-Communicable Diseases Among Ghanaian Adults: A Survey at a Secondary Healthcare Level, PLoS One. (2023) 18, no. 2, e0281310, 10.1371/journal.pone.0281310, 36753484. PMC9907821 36753484

[19] Gosdin L. , Sharma A. J. , Tripp K. , Amoaful E. F. , Mahama A. B. , Selenje L. , Jefferds M. E. , Martorell R. , Ramakrishnan U. , and Addo O. Y. , A school-Based Weekly Iron and Folic Acid Supplementation Program Effectively Reduces Anemia in a Prospective Cohort of Ghanaian Adolescent Girls, Journal of Nutrition. (2021) 151, no. 6, 1646–1655, 10.1093/jn/nxab024, 33758915. PMC8172428 33758915

[20] Tandoh M. A. , Agyemang W. O. , Brago E. T. , Attu S. S. , and Domfeh E. A. , Prevalence and Risk Fctors of Anaemia Among Women of Reproductive Age in a Ghanaian University, Journal of Science and Technology. (2024) 42, no. 4, 80–93, 10.4314/just.v42i4.8.

[21] Dziwornu E. , Aguadze E. , Dordoye E. , and Sarfo J. O. , Sleep Disorders and Patterns among Medical Students at the University of Health and Allied Sciences, Ghana, European Journal of Contemporary Education. (2022) 11, no. 4, 1098–1104, 10.13187/ejced.2022.4.1098.

[22] Mbous Y. P. V. , Nili M. , Mohamed R. , and Dwibedi N. , Psychosocial Correlates of Insomnia Among College Students, Preventing Chronic Disease. (2022) 19, 10.5888/pcd19.220060. PMC9480843 36108290

[23] Ruivo Marques D. , Allen Gomes A. , and de Azevedo M. H. P. , Perceived Causes of Sleep Problems in Higher Education Students: An Exploratory Study, Psychological Reports. (2026) 129, no. 4, 2908–2930, 10.1177/00332941241299730. PMC13287506 39520240

[24] Çakar E. , Gümüştakım R. Ş. , and Kuş C. , Evaluation of The Knowledge and Awareness of Anemia in Medical Faculty Students and Family Physicians, Kahramanmaraş Sütçü İmam Üniversitesi Tıp Fakültesi Dergisi. (2022) 17, no. 2, 140–148, 10.17517/ksutfd.897826.

[25] Misra S. , Knowledge, Attitude and Practices (KAP) Towards Anaemia Among Female University Students in MALAYSIA: A Cross-Sectional Survey, Malaysian Journal of Nutrition. (2022) 28, no. 1, 91–104.

[26] Atitsogbey P. , Kyereh E. , and Steiner-Asiedu M. , Nutrition Education Improves Households′ Knowledge, Attitudes, and Practices Towards Consuming Indigenous Green Leafy Vegetables and Legumes in Accra, Ghana, Scientific African. (2025) 28, e02710, 10.1016/j.sciaf.2025.e02710.

[27] Buboltz W. , Jenkins S. M. , Soper B. , Woller K. , Johnson P. , and Faes T. , Sleep Habits and Patterns of College Students: An Expanded Study, Journal of College Counseling. (2009) 12, no. 2, 113–124, 10.1002/j.2161-1882.2009.tb00109.x.

[28] Zegeye B. , Anyiam F. E. , Ahinkorah B. O. , Ameyaw E. K. , Budu E. , Seidu A.-A. , and Yaya S. , Prevalence of Anemia and Its Associated Factors Among Married Women in 19 Sub-Saharan African Countries, Archives of Public Health. (2021) 79, no. 1. 10.1186/s13690-021-00733-x PMC8628470 34839822

