# Supplementary material for: High Anemia Awareness Does Not Mitigate Insomnia Risk in Ghanaian University Students: A Cross‐Sectional Study
Source: Biomed Res Int. 2026 Oct 8;2026:8968775. doi: 10.1155/bmri/8968775 (PMC13650385; doi:10.1155/bmri/8968775)
Supplement: Supplementary file 1 [file BMRI-2026-8968775-s001.docx]

**Title of Study:** Exploring Anaemia and Insomnia Prevalence, and the Role of Prevention Knowledge in Ghanaian University Students: A Cross-Sectional Study

**CONSENT STATEMENT**

I, ………………………………………………………., voluntarily agree to participate in the research study titled “**Exploring Anaemia and Insomnia Prevalence, and the Role of Prevention Knowledge in Ghanaian University Students: A Cross-Sectional Study**” conducted by a group of final year Medical Laboratory Science students from the University of Cape Coast (UCC). I understand the purpose of the study, which is to explore knowledge attitudes and practices regarding anaemia and its prevention as well as insomnia status of participants.

I acknowledge that my participation is entirely voluntary, and I have the right to withdraw at any time without any consequences. I have been given the opportunity to ask questions and have received satisfactory answers. I understand that the information provided by me will be used solely for research purposes, and will be kept confidential.

By proceeding with this questionnaire, I affirm that I am at least 18 years old and give my informed consent to participate in this research study.

.…………………….

Participant’s signature

____/_____/__________

Date

**SECTION 1**

Socio-Demographic information

1. Age (years), [] 18-20 []20-22 []23-25 []>25

2. Gender; [] Male [] Female

3. Marital status; [] Single [] Married [] Other

4. Religion; [] Islam [] Christianity [] Others

5. Level of student; []100 []200 []300 []400 []500 []600

6. Faculty; [] health related [] non-health related

7. Program of Study …………………………….

8. Chronic health status; [] No [] Yes

9. History of anaemia in past three (3) years [] No [] Yes

10. Do you take alcohol? [] Yes [] No

11. Any history of blood transfusion? [] Yes [] No

12. Have you ever taken iron / multivitamin supplement? [] Yes [] No

**SECTION 2**

General Knowledge Assessment of Participants on Anaemia

1. Blood has a reduced ability to carry oxygen when anaemia is present. [] Yes [] No [] Not sure
2. Hypotension and loss of appetite may be some symptoms of anaemia. [] Yes [] No [] Not sure
3. Malabsorption and poor diet intake are some causes of anaemia. [] Yes [] No [] Not sure
4. Aged people are mostly at risk of anaemia. [] Yes [] No [] Not sure
5. Iron deficiency can cause anaemia. [] Yes [] No [] Not sure
6. When untreated, anaemia could lead to coma. [] Yes [] No [] Not sure
7. Pica occurs as a result anaemia. [] Yes [] No [] Not sure
8. Athletes are mostly at risk of anaemia. [] Yes [] No [] Not sure

**SECTION 3**

**Assessment of Misconceptions of Participants**

1. Anaemia is a spiritual disorder. [] Yes [] No [] Not sure
2. Anaemia is contagious or transmissible by body contact. [] Yes [] No [] Not sure
3. Sickle cell anaemia affects Males more than Females. [] Yes [] No [] Not sure
4. Men are more prone to anaemia compared to women. [] Yes [] No [] Not sure
5. Consistently eating thrice, a day can prevent anaemia. [] Yes [] No [] Not sure
6. Pregnant women who eat well require iron and folic acid supplement [] Yes [] No [] Not sure
7. Anaemia is a disorder of the liver.	[] Yes [] No [] Not sure
8. Eating early everyday can prevent anaemia. [] Yes [] No [] Not sure

**SECTION 4**

**Assessment of Anaemia Management and Prevention**

1. Blood transfusions can be used to manage anaemia. [] Yes [] No [] Not sure
2. Eating balanced diet and iron rich diets can prevent anaemia. [] Yes [] No [] Not sure
3. Intake of green vegetables, beans and poultry provides folic acid that can prevent anaemia.

[] Yes [] No [] Not sure

4. Treatment of worm infestation can help in anaemia management. [] Yes [] No [] Not sure
5. Having regular medical checkup helps to monitor and manage anaemia. [] Yes [] No [] Not sure
6. Preventing malaria infection can help to prevent anaemia. [] Yes [] No [] Not sure
7. Iron and folic acid supplements can help manage anaemia in females. [] Yes [] No [] Not sure
8. Avoiding intake of non-nutritious substances like clay helps prevent anaemia. [] Yes [] No [] Not sure
9. Avoiding excessive alcohol intake can help prevent anaemia [] Yes [] No [] Not sure
10. Preventing kidney disorders can help prevent anaemia [] Yes [] No [] Not sure

**SECTION 5**

**Attitudes and Practices towards Anaemia Prevention and Management**

1. Do you consult a Physician when you have symptoms like dizziness and shortness

of breath?	[] Yes [] No

2. Do you seek health education on anaemia? [] Yes [] No
3. I prefer to eat a heavy meal once or twice a day due to financial issues. [] Yes [] No
4. Any health worker can prescribe anaemia medication for me. [] Yes [] No
5. Do you think the food you eat has nothing to do with anaemia? [] Yes [] No
6. I’d rather eat vegetables than take supplements [] Yes [] No
7. There’s no need to follow up on my health once I’ve been prescribed medication. [] Yes [] No
8. I don’t see the importance of sleeping under insecticide treated bed net. [] Yes [] No
9. Do you do self-management of anaemia most of the times. [] Yes [] No

INSOMNIA SEVERITY INDEX

The Insomnia Severity Index has seven questions. The seven answers are added up to get a total score.

For each question, please CIRCLE the number that best describes your answer.

Please rate the CURRENT (i.e. LAST 2 WEEKS) SEVERITY of your insomnia problem(s).

| Insomnia Problems | None | Mild | Moderate | Severe | Very Severe |
| --- | --- | --- | --- | --- | --- |
| 1.Difficulty in falling asleep | 0 | 1 | 2 | 3 | 4 |
| 2. Difficulty staying asleep | 0 | 1 | 2 | 3 | 4 |
| 3. Difficulty waking up too early. | 0 | 1 | 2 | 3 | 4 |

4. How SATISFIED/DISSATISFIED are you with your CURRENT sleep pattern?

Very Satisfied	Satisfied	Moderately	Satisfied Dissatisfied	Very Dissatisfied

0	1	2	3	4

5. How NOTICEABLE to others do you think your sleep problem is in terms of impairing the quality of your life?

Not at all

Noticeable	A Little	Somewhat	Much	Very Much Noticeable

0	1	2	3	4

6. How WORRIED/DISTRESSED are you about your current sleep problem?

Not at all

Worried	A Little	Somewhat	Much	Very Much Worried

0	1	2	3	4

7. To what extent do you consider your sleep problem to INTERFERE with your daily functioning (e.g. daytime fatigue, mood, ability to function at work/daily chores, concentration, memory, mood, etc.) CURRENTLY?

Not at all

Interfering	A Little	Somewhat	Much	Very Much Interfering

0	1	2	3	4
